# Studying Medicine and being a doctor in Spain

**DOI:** 10.15694/mep.2018.0000276.1

**Published:** 2018-12-07

**Authors:** Joaquín García-Estañ

**Affiliations:** 1 Universidad de Murcia

**Keywords:** Access to University, Admission to Medicine, Numerus Clausus, Medical Specialization, Medical School

## Abstract

This article was migrated. The article was marked as recommended.

In Spain, and probably around the world, the degree of Medicine is one of the most appreciated studies by the students. Not only the influence of job prospects, better than in other University careers, but also the study of a scientific career with many specialization areas or the joy felt by doctors when they manage to help a very sick patient, are some reasons to undertake the adventure to become a doctor. Since the demand to study Medicine is so high, it is not strange that most people with interest stay out of the process, since, at least in Spain, only the high school students with the best records are able to enter into a School of Medicine. But becoming a doctor is more than the study of a University Degree for 6 years. They need also a postgraduate specialization (MIR), in Hospitals of the National Health System, as a necessary step in order to be able to practice Medicine, either in public or private institutions. However, this road is not free from problems since there has been a decrease in the number of MIR specialization places offered without a similar reduction in the number of undergraduate positions. Moreover, the number of medical schools has not stopped growing and places Spain as one of the countries with the highest ratio of Medical Schools per inhabitant. This situation can lead to a scenario similar to that experienced in the 80s and 90s, where we witnessed an increase in the number of Specialist Physicians without Official Title (called MESTOS). As of November 2018, around 4,000 medical graduates cannot access specialized health training and may be forced into unemployment or emigration.

## Introduction

**Figure 1.**
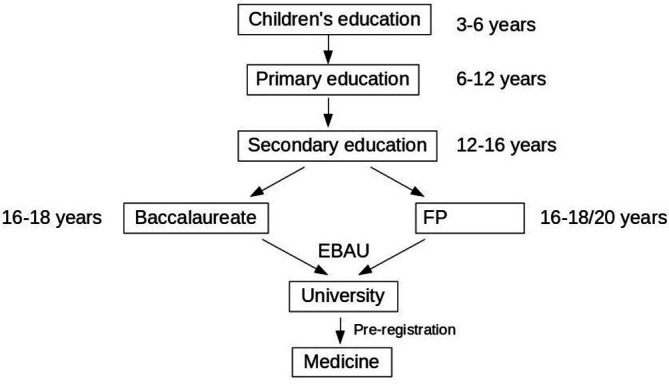
The Spanish educative system.

The Spanish Educational System determines that compulsory education extends from 6 to 16 years. Once the primary education is done and after going through secondary education, the student can choose to continue their education with the Baccalaureate in a High School (Bachillerato, BAC) or in a Vocational Training school. Once this is finished, they can apply for entrance to University, where, in general, young people enters at 18 years of age ( figure 1).

The law that regulates admission to the University studies (Ministerio de Educación, Cultura y Deporte, 2014) establishes that, although each University may have specific requirements for enrollment in the desired School, those interested must have a Baccalaureate’s degree from the Spanish Educational System or equivalent (European Baccalaureate diploma or International Baccalaureate). Therefore, it is indirectly understood that it is necessary to be at least 17/18 years of age to enter the University. In Spain, around 300,000 people enter each year to Universities (Ministerio de Educación, Cultura y Deporte, 2016) to study a Degree in one of five main knowledge areas. They are (data from the course 15-16) Social and Legal Sciences, a 46% of the total, Engineering and Architecture (20%), Arts and Humanities (13%), Health Sciences (15%) and Sciences (7%). Of these 45,000 that enter to a degree in Health Sciences, around 7,000 enter in one of the more than 40 Medicine Schools in Spain. However, there are estimates that more than 15,000 people wishing to study Medicine cannot be admitted.

## Access and admission

There are two important concepts that we should know, **access** and **admission**. Access to the University System and Admission to a specific University and to a Degree. The term “Access” thus determines the process to enter the University System and is valid for the whole country. Although the access tests (traditionally called Selectivity) are carried out in all Autonomous Regions, the score obtained is valid for the entrance at any University in Spain. This is because of the concept of “Single or Common District”, whereby students can decide where to study. The access is governed by the Education Law of 2001, reformed in 2008, which indicates that the “access to the Spanish University.. will be made with full respect for fundamental rights and the principles of **equality, merit and ability**” ( Ministerio de la Presidencia, 2008).

### Access to the University from Baccalaureate and Vocational Training

The examination for access, organized by the Universities in conjunction with their Autonomous Governments, is called Evaluation of Baccalaureate for the Access to the University (EBAU) and it is composed of several exams of mandatory matters of the last year of Baccalaureate (it is called the general stage) and at least two elective matters (or voluntary stage), that belong to the five main knowledge areas of the students wish to study. For instance, those wishing to study Medicine, belong to the Baccalaureate of Health Sciences and they should study Biology, Earth Sciences, Physics, and Chemistry ( Ministerio de la Presidencia, 2008).

The general stage of EBAU is composed of general knowledge matters such as History of Spain, Spanish Language and Literature, Foreign Language and another one depending on the modality of their Baccalaureate, i.e. Mathematics as in Health Sciences or Latin in Humanities. Each of these exams is graded between 0 and 10 points, and the general phase score will be the average of these four exams expressed with three decimals (no minimum is required). If this grade reaches at least 4 points, the University access note is calculated, with a weight of 40% for the general stage and 60% for the average grade obtained in the Baccalaureate. Thus, the maximum grade that can be obtained, the University access note, is 10. The access to the University is granted with at least 5 points. The voluntary stage allows the improvement of these scores up to 14 points. In this phase, the students take as many exams are desired and the best two of them are added to the score of the general phase. A minimum of 5 should be obtained and these grades are weighted usually by a factor of 0.2. Thus, two exams with a maximum of 10 points give the students 4 additional points. The results of the general stage plus the scores of the voluntary stage now make the **Admission Score**, that the students use to apply, in any University, for their desired Degrees ( Ministerio de la Presidencia, 2008).

### Admission to Medicine

The Degree of Medicine can be studied today (2018) in 42 Schools (Faculties) of Medicine, all of them affiliated to different Universities. Most of them are public Centres (31), and 11 of them are private ( table 1). Public Universities are funded by the State through the Autonomous Regions, and they charge an amount as tuition, that is regulated by the Autonomous Governments. All the degrees have 60 ECTS per year, for 6 years, 360 ECTS. One ECTS is composed of 10 classroom hours and 15 hours of student workload.

**Table 1.** Maximum number of students admitted to Schools of Medicine in Spain in 2018 (numerus clausus), together with their cut-off grade and tuition (€).

		Numerus Clausus	Cut-off Score	Tuition (€)
**Autonomous Region**	**Public Universities**			
ANDALUCÍA	CADIZ	155	12.483	757
	CORDOBA	130	12.623	757
	GRANADA	253	12.750	757
	MALAGA	165	12.631	757
	SEVILLA	291	12.725	757
ARAGÓN	ZARAGOZA	180	12.450	1,403
	HUESCA	45	12.301	1,403
ASTURIAS	OVIEDO	153	12.370	1,355
BALEARES	MALLORCA	60	12.446	1,388
CANARIAS	LA LAGUNA	130	12.546	1,046
	LAS PALMAS	135	12.604	1,046
CANTABRIA	CANTABRIA	119	12.183	964
CASTILLA LA MANCHA	ALBACETE	125	12.801	1,132
	CIUDAD REAL	71	12.745	1,132
CASTILLA Y LEON	SALAMANCA	180	12.321	1,815
	VALLADOLID	180	12.210	1,815
CATALUÑA	AUTONOMA BARCELONA	320	12.202	2,372
	BARCELONA	259	12.362	2,372
	GIRONA	80	12.140	2,372
	LLEIDA	110	12.110	2,372
	POMPEU FABRA	60	12.422	2,513
	ROVIRA I VIRGILI	125	12.080	2,372
VALENCIA	JAUME I	80	12.780	1,389
	MIGUEL HERNANDEZ	130	12.851	1,389
	VALENCIA	320	12.990	1,389
EXTREMADURA	EXTREMADURA	120	12.315	1,111
GALICIA	SANTIAGO	360	12.286	836
MADRID	ALCALÁ	120	12.747	1,609
	+ Army	25	12.675	0
	AUTONOMA MADRID	240	13.110	1,609
	COMPLUTENSE	295	12.871	1,609
	REY JUAN CARLOS	150	12.793	1,609
MURCIA	MURCIA	200	12.819	1,007
PAIS VASCO	PAIS VASCO	325	12.331	1,225
**Total Public Universities**		**5,691**	**12.532**	**1,395**
				
	**Private Universities**			
CATALUÑA	INTERNACIONAL CATALUÑA	100	N.A.	13,920
	VIC	80	N.A.	13,791
MADRID	ALFONSO X EL SABIO	132	N.A.	20,254
	EUROPEA DE MADRID	220	N.A.	20,440
	FRANCISCO DE VITORIA	120	N.A.	17,000
	C.E.U. SAN PABLO	160	N.A.	20,240
MURCIA	UCAM	99	N.A.	11,395
NAVARRA	NAVARRA	200	N.A.	15,204
VALENCIA	CATÓLICA VALENCIA	120	N.A.	12,490
	CEU-CASTELLON	70	N.A.	17,000
	CEU-VALENCIA	50	N.A.	21,000
**Total Private Universities**		**1,351**	**N.A.**	**16,612**

Source: Ministry of Education, references 10 and 11.

The admission to Medicine is governed in public Universities by the general principles of **equality, merit and ability**. Usually, they maintain a high level of transparency, although always with varying degrees ( Fundación Compromiso y Transparencia, 2016). The degree of Medicine was the first in the Spanish University to introduce a fixed number of available positions, what we call today “numerus clausus” ( Jefatura del Estado, 2007). This which means that all Schools have a maximum number of students they can admit. This figure is approved by the Autonomous Governments first, and finally by the Conference of University General Policy of the Ministry of Education. The main reason to establish numerus clausus was the admission of a number of students who could be adequately managed by the Universities, not only in theoretical classes but more importantly during hospital training trying, to avoid overcrowding thus allowing an adequate training. During the last years, the creation and approval of private schools of Medicine is risking the hospital training of students from public Universities, since some governments insist in that public hospitals can be shared by several Universities, private and public. There are some voices that argue that numerus clausus only exists to benefit private Universities, especially in Medicine, since the demand to become a doctor exceeds two or three times the positions offered by all the Universities ( Martí, 2016; Foro de la Profesión Médica de España, 2014).

**Figure 2.**
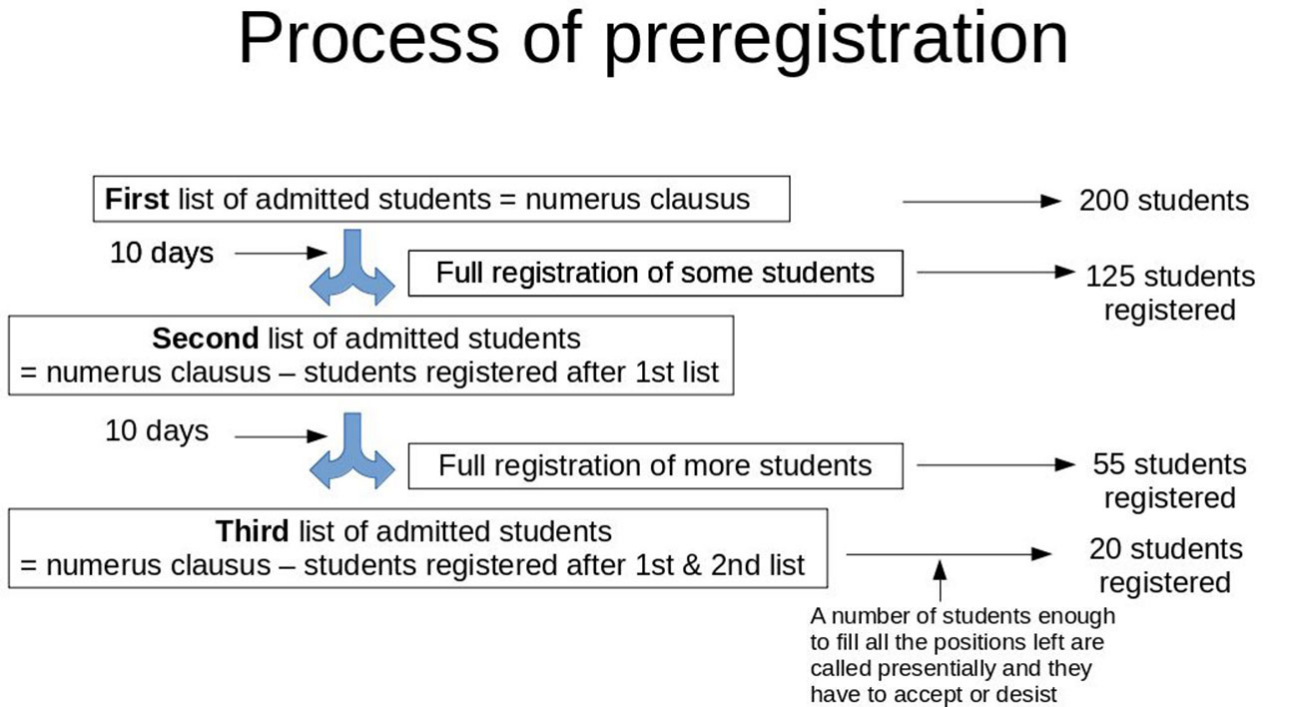
Procedure of admission to a Public Medical School in Spain.

In order to admit students to degrees with numerus clausus, all the Universities organize a previous step to full registration, called **preregistration**, in which they receive applications from students all over the country, since as we said, all the public Universities belong to the “common district”, i.e. they can study in a different University to that which organized their access exam. This process has been widely criticized ( García-Crespo, 2008; García-Crespo, 2009) since each University organizes this process without coordination with the rest, which forces many students to preregister at many Universities in order to ensure their admission to at least one of them. Of course, many students are admitted in several Universities and several lists of admitted students are published during several months (June to October) until all positions are awarded ( figure 2). In many cases, the academic course starts at the beginning of September when the class of first year is now complete yet.

Preregistration, then, is a system that organizes all the applications regarding their Admission Score, thus only the students with the best scores are admitted ( figure 2). The preregistration system has allowed also the establishment of a ranking of Medicine schools based on these scores, specifically on the score of the last student admitted (Ministerio de Educación, Cultura y Deporte, 2018). This cut-off note (nota de corte in Spanish) is even used by the Ministry of Education in its web page in order to give information about the difficulty of the entrance to any given School. The cut-off grade average in the Spanish Public Universities in 2018 is 12.5 ( table 1). Thus, if the admission grade of a student is below this figure, it may be difficult to enter Medicine in the desired school, forcing the student to apply to several of them. In the past, there was an attempt to establish a national system of preregistration, similar to that organized for the allocation of MIR positions (see later), but it was rejected by some autonomous communities on the fear of losing autonomy. This system was presented by the National Association of Medical Deans in order to prevent many families from traveling all over Spain and offer a modern, online, fair and transparent way of allocating the seats to Medical Schools ( García-Estañ, 2010; Universidad de Murcia, 2010).

The admission system also reserves some places for special students. Thus, the total number of places that are offered in each Degree and Center are distributed according to the percentages legally established for each reservation quota. These are:

1. Two percent (2%) of the places for applicants who are already in possession of an official University degree or equivalent.
2. Three percent (3%) of the places in favor of applicants who have passed the entrance exam to the University for over 25 years of age.
3. Two percent (2%) are reserved for applicants over 40 or 45 years of age, distributed at a rate of one percent (1%) for people over 40 years old and another one percent (1%) for adults over 45 years.
4. Five percent (5%) of the places available for applicants who, meeting the academic requirements of access, have recognized a degree of disability equal to or greater than thirty-three percent (33%), as well as for students with special and permanent educational needs.
5. In the case of high level and high-performance athletes, a three percent (3%) of places.

### The cost of study Medicine in Spain

The tuition from public Universities ranges from less than 1,000 up to 3,000 € per academic year ( table 1). The public Universities that offer these studies more economically, according to data from 2017-2018, are those of Andalucía (Cádiz, Córdoba, Granada, Málaga and Sevilla) with a credit price of 12.62 euros, the University of Santiago de Compostela in Galicia (13.93 euros) and the University of Cantabria (16.07). At the other extreme, the most expensive public ones are in Cataluña, where the University of Barcelona, Pompeu Fabra University and the Autonomous University have a price per credit of 39.53 euros. Thus, the price per course is set at 757.20 euros in Andalusia and 2,371.80 euros in Cataluña ( table 1).

### Admission to Medicine in Private Universities

Private Schools have their own mechanisms of admission, and most of them perform an additional examination to their interested students, together with a personal interview. Some Universities share their entry exams online (Universidad CEU, 2018). In general, there is not much transparency in these processes ( Fundación Compromiso y Transparencia, 2016). Their tuition charges are also much more important than those of public universities ( table 1).

### Other admission entries to Medicine

Those who want to study Medicine, once the selectivity is finished, can also access the studies through the Army. The Military School of Health offers the possibility of studying medicine and, simultaneously, receiving technical and military training. In this way, the student is trained as a career military officer at the Officers’ Scale of the Military Health Corps, a fundamental specialty in Medicine. The University Center of Defense is a higher education body of the Ministry of Defense, attached to the University of Alcalá de Henares (Madrid). It offers 30 places to access the undergraduate studies without prior University qualification, of which 25 are direct income and 5 internal promotion. The cut-off grade in the course 2017-2018 was very similar to those of the public universities and the studies are completely free. Technical and military training is mandatory to practice as a military doctor, but for six years you can access this training through the Baccalaureate degree and selectivity. All the expenses of the training are borne by the State. The students have also maintenance and lodging. In addition, they receive a remuneration for being considered members of the military corps. Once the studies are completed, the doctor acquires the commitment to remain in the army for twelve years. In the case of not complying with this easement, the graduate must compensate the State financially for the training received.

### Is it time for a change?

Each year thousands of students intend to study medicine, but many remain in the attempt despite having excellent records. Probably many of them could be good doctors if they had the chance. Conversely, the entry of students selected solely for their excellence in high school, without taking into account other factors such as vocation care, does not warrant that we would have excellent doctors. In fact, Universities are allowed to establish their own admission rules to University degree courses using only the criterion already mentioned of the final mark obtained in High School, as it is the case up until now, or, this is new, they can set specific admission procedures, that until now, only private Universities use ( Ministerio de la Presidencia, 2008). Some authors ( Palés, 2018) have suggested that since medical practice requires the highest standards of professional and personal conduct, we should also evaluate candidates in humanistic competencies and other personal qualities such as communication, compassion and commitment to service, which can already be observed in many students but not in others. Clearly, the implementation of such initiatives is by no means easy and requires a long and detailed analysis of medical education and university experts to ensure that the procedures adopted are acceptable and satisfactory to all, maintaining the features of the access to public education systems.

## Access to postgraduate training: becoming a specialist

Every year, in the first days of September, the Ministry of Health publishes in the State Official Bulletin ( Ministerio de Sanidad, Consumo y Bienestar, 2018) the offering of positions to enter the process of specialized health formation, i.e., the positions offered to become a Resident in one of more than 50 medical specialties. This process is known as the MIR exam since MIR is the acronym designed for Medico Interno Residente (Internal Resident Doctor). The last one published (September 2018) offered 6,797 positions for medical graduates as well as 267 for graduates in Pharmacy, 1.092 for nursing, 22 for Chemistry, 49 for Biology and 141 for Psychology, and 34 for Physics. The whole process is of a selective nature, and organized nationally, with tests taking place in most province capitals. The exam date is usually in February of the following year, and the selected graduates will start their training around the last days of May, almost one year after graduation from the medical school. Table 2 shows the total specialization positions offered according to the medical specialty. Family and Community Medicine is by far the specialty with more positions offered, although it has been described its lack of success among medical graduates ( Freire *et al.*, 2015).

**Table 2.** Medical specialities positions offered for postgraduate specialization in 2018.

Aesthetic and Reconstructive Plastic Surgery	40
Allergology	61
Anesthesiology and resuscitation	344
Angiology and vascular surgery	35
Cardiology	168
Cardiovascular Surgery	24
Clinical Analysis	88
Clinical Biochemistry	43
Clinical Neurophysiology	40
Clinical Pharmacology	16
Digestive system	161
Endocrinology and Nutrition	81
Family and Community Medicine	1914
General and digestive surgery	198
Geriatrics	66
Hematology and Hemotherapy	123
Immunology	29
Intensive medicine	163
Internal Medicine.	334
Medical Oncology	120
Microbiology and Parasitology	80
Nephrology	97
Neurology	129
Neurosurgery	45
Nuclear medicine.	45
Obstetrics and Gynecology	266
Ophthalmology	176
Oral and Maxillofacial Surgery	32
Orthopedic Surgery and Traumatology	243
Otorhinolaryngology	83
Pathological anatomy	104
Pediatric surgery	23
Pediatrics and Specific Areas	433
Physical Medicine and Rehabilitation	99
Pneumology	115
Preventive medicine and public health	69
Psychiatry	248
Radiation Oncology	62
Radiodiagnosis	229
Rheumatology	56
Surgical Medical Dermatology and Venereology	94
Thoracic surgery	26
Urology	105
Work Medicine	65
Total	6972

https://www.boe.es/boe/dias/2018/09/14/pdfs/BOE-A-2018-12537.pdf).

The selective tests for those who intend to access the places of Medicine, Nursing, Pharmacy and other degrees consist of an exercise of 225 multiple choice questions, different for each of these tests, whose duration is five hours. The individual total score of each applicant in the selective test is obtained from the sum of the one obtained in the exercise of multiple answers and that assigned to the academic merits. The score of the multiple answer exercise will be obtained taking into account the correct but also the incorrect answers in the following way: each valid answer receives a value of three points, and one point is subtracted for each of the incorrect answers. Logically, the unanswered questions are not assessed. After all the tests have been scored, the arithmetic mean of the ten maximum individual assessments is obtained and given a value of 90 points. Then, the final score of the exercise for each applicant is obtained multiplying by 90 the particular valuation of it and dividing the product by the arithmetic mean referred to previously. The exam is considered passed, and the applicants will be able to participate in the allocation of a place of MIR training when the applicant obtains a minimum score that is equal or greater than 35% of the arithmetic mean of the ten best ratings of the exercise. This is called the cutoff score. Finally, the academic record of the graduate is also evaluated (the average grade of all the matters in the degree) and, where appropriate, the Doctor’s degree. This is done in a similar manner. After assessing all the academic records of all applicants who would have performed the exercise, the arithmetic mean of the ten highest ratings is obtained and this is given the value of 10 points. The score of the academic merits of each applicant is obtained by multiplying by 10 its particular valuation and dividing the product by the arithmetic mean obtained previously. The final grade of the applicant thus calculated represents, therefore, 90% of the test and 10% of the academic record.

Not only the recently graduated doctors are able to take the MIR exam, although they are usually more than 90%. Doctors that obtained a position in a previous year but have not finished residency may also resign and take the MIR test again. This is usually done to improve the position obtained and/or to be able to choose a different specialty. An estimated 3% of applicants take this path. Another 3% of applicants is estimated to concur again to MIR exam after finishing a residency, to obtain a different specialty. There is also a quota for foreign graduates. Not for graduates from countries of the European Union (EU), who are treated as national students, but for non-EU graduates, mostly from Latinamerica. This quota is 4%, meaning that a maximum of 4% of the specialization positions can be taken by non-EU doctors. Doctors with a recognized disability also have a 7% of the offered specialties reserved. For the first time, in the MIR 2012, the Ministry reserved some places (7%) for doctors with a disability higher than 33%. The applicant with a disability can choose the place and specialty, according to the order number obtained, which he considers compatible with his particular situation, without prejudging the result, positive or negative, of the initial mandatory medical examination to which all the residents (with or without disabilities), upon joining the adjudicated place. The reserved places in the disability shift that are not covered are reassigned to the general quota.

## Towards a change on MIR exam

The MIR exam is regarded as a good method to organize the postgraduate education according to the principles of equality, merit and ability, similar to those governing the entry to the graduate studies. However, the exam is essentially the same for the last 40 years and there are several and important critics to it ( Vázquez et al., 2008; Aspa-Marco and Rodríguez de Castro, 2010; Freire et al., 2015; Lobato et al., 2015). Maybe the most serious one is the opinion that it negatively influences the graduate formation in Medical Schools. Rozman (2005) has written that “being exclusively cognitive, seriously conditions the degree studies. The paradox occurs that the faculties of medicine do not even prepare their students for this exam since these usually go to academies specialized to overcome more easily the test”. We all know that students start preparing the MIR exam well in advance of their graduation, in some cases, academies start the process in the first year of medical studies! In most cases, graduates spend one year of their lives learning MIRicine (invented word coming from the fusion of MIR and Medicine) but not Medicine, much less mastering the clinical competences they should start using during residency. Medical schools are responsible also, since they allow academies to run freely among their students ( Aspa-Marco and Rodríguez de Castro, 2010; Lobato et al., 2015). A solution to this problem, consists in a modification of the MIR exam. Even the legislators thought of a solution more than 15 years ago. The Law of Organization of the Health Professions (Jefatura del Estado, 2003) proposed to include in the MIR exam, together with the cognitive aspects, the examination of clinical skills and communications, a kind of OSCE that all graduates would have to pass. Although most medical schools in Spain now examine their students of a 20 station-OSCE ( García-Estañ, 2013), no efforts have been made by authorities to implement measures that reinforce the acquisition of clinical and communication skills before entering the postgraduate formation. In conclusion, the method for selecting candidates to residency places currently used in Spain, which relies mainly on testing theoretical knowledge, should be changed for an alternative methodology taking into account the students performance and assessing his/her ability for clinical contextualization of knowledge and level of clinical competence ( García-Estañ, 2013; Aguayo-Albasini and García-Estañ, 2016).

## Medical demography in Spain

**Figure 3.**
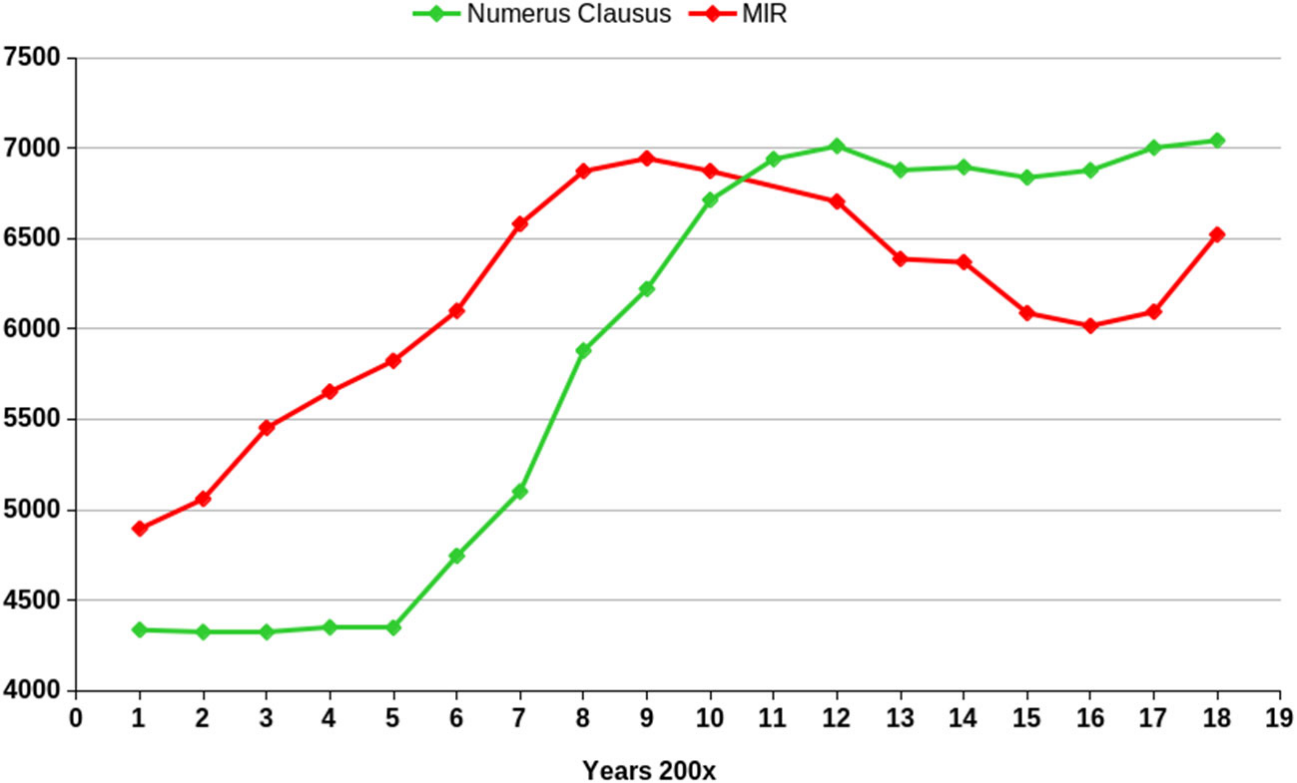
Numerus clausus in Medical Schools and Specialization positions offered since the beginning of the XXI century in Spain.

A comprehensive database regarding medical demography is maintained by the General Council of Medical Doctors Association ( OMC, 2018) and a recent report has been published. Since the beginning of the XXI century, public Universities have increased their ‘numerus clausus’ by more than 30%, going from around 4,500 places in 2001 to 5,700 in September 2018. The places of the Private Universities have had an accumulated growth in this same time period of 210%, going from 230 places in 2001 to 1,351 in the current academic year. More than 7,000 places in all the Spanish Faculties for the academic year 2017-18. Figure 3 shows the evolution of numerus clausus and MIR positions since 2001.

**Figure 4.**
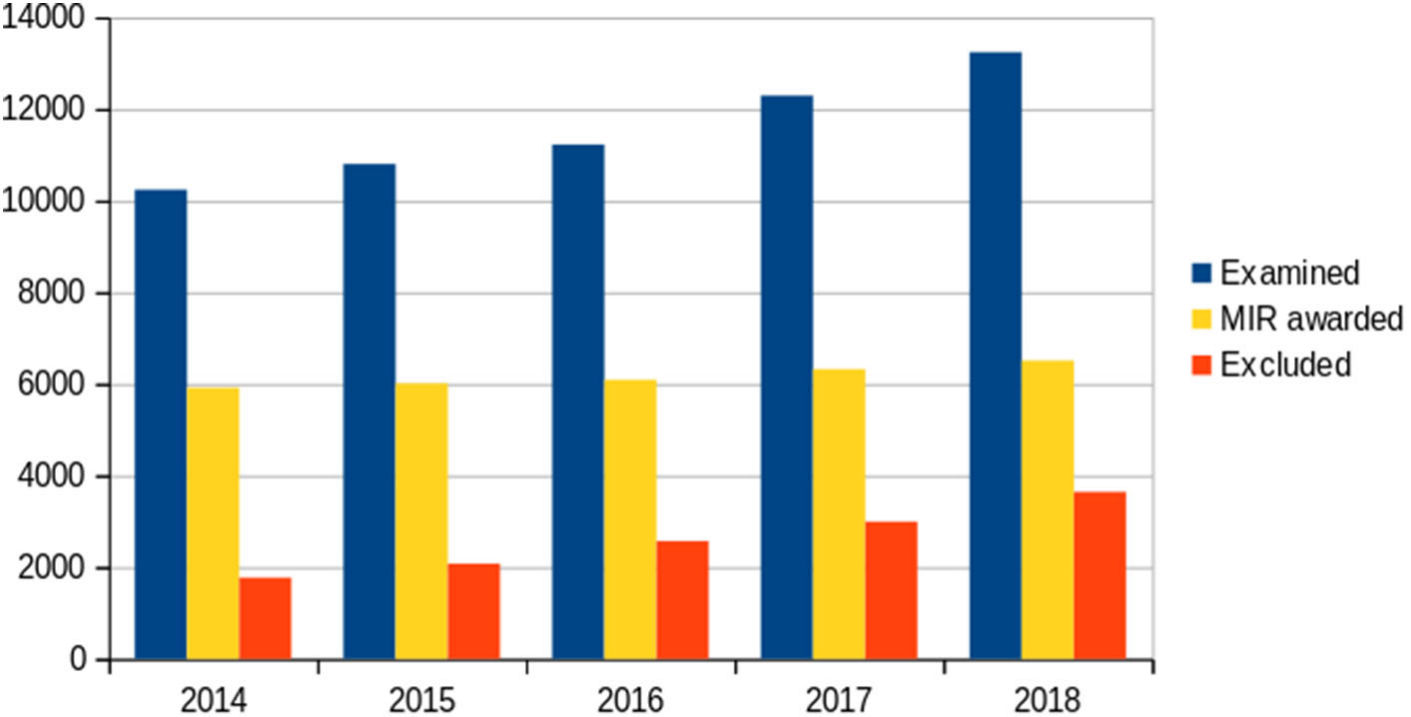
Number of specialization positions awarded to medical graduates (yellow bars) from the total number of graduates that took the MIR exam (blue bars). Red bars show the number of graduates excluded from specialization (3,650 in 2018). Data are from both Spanish and non-Spanish medical graduates.

Two different lines can be observed, reflecting the lack of coordination between Education (numerus clausus) and Health (MIR) authorities. While MIR places were increasing at the beginning of the century, numerus clausus was maintained well below those numbers. However, also during those years, all of these MIR positions were always awarded, since many foreign non-UE doctors entered the process with no limit, as the maximum of 4% was implemented later. Then, in 2006 the pressure on existing Medical Schools and the opening of new ones started to elevate numerus clausus until reaching a maximum in the 2012-13 academic year. It was the course with the highest number of places awarded in the history of the medical career in Spain, until now in September 2018. However, almost simultaneously, the economic crisis hit severely Spain and we lost many of these MIR positions, thus creating a surplus of recent graduates with no possibility of obtaining a MIR position to specialize ( figure 4).

**Figure 5.**
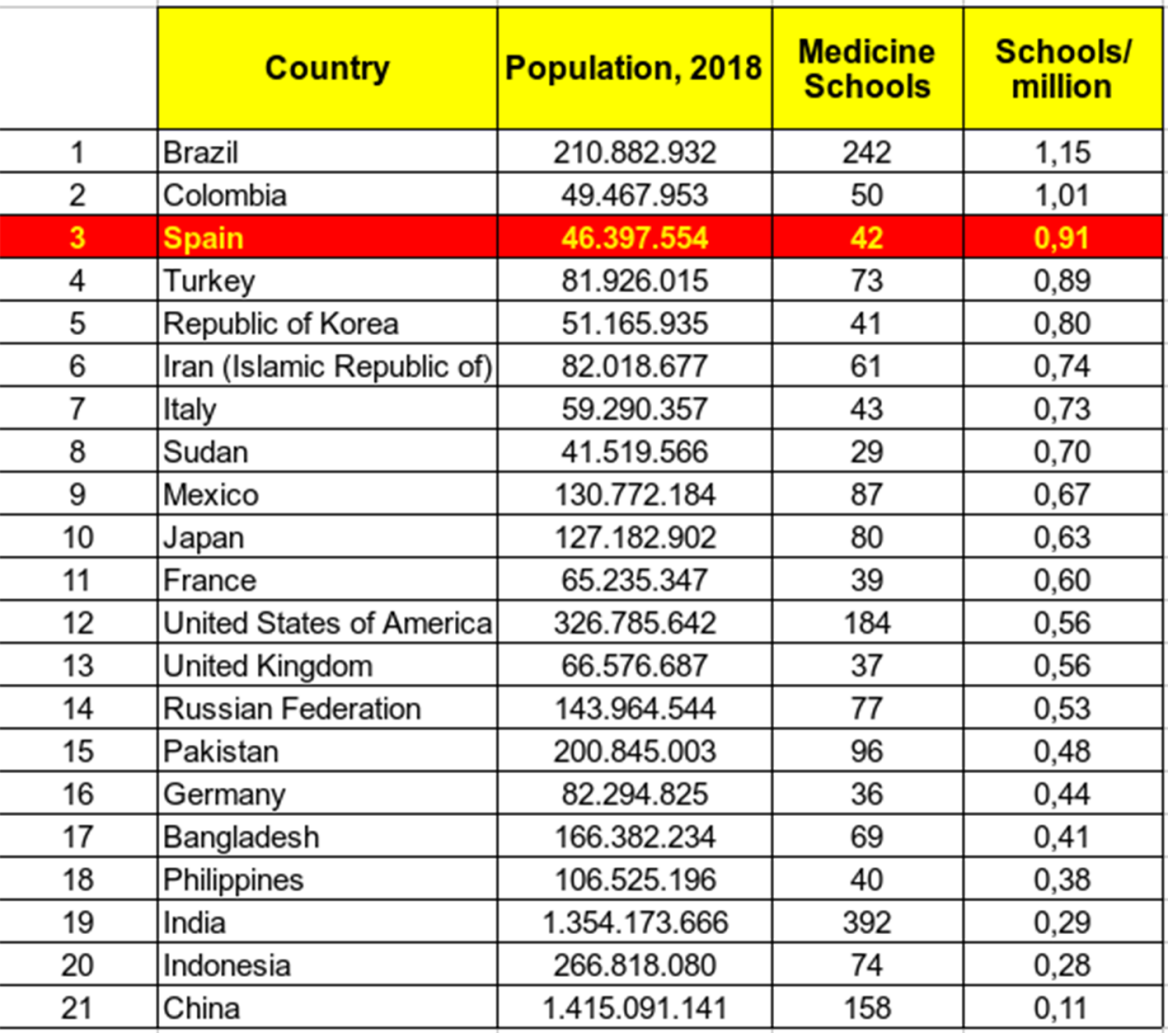
World ranking of countries ordered by Medicine Schools to population ratio. Sources: obtained on July 5, 2018 from http://worldpopulationreview.com/countries (Population) and Medicine schools: http://avicenna.ku.dk/database/medicine/.

According to the report “Health at a Glance 2017” ( OECD, 2017), published at the beginning of November 2017 by the OECD (Organization for Economic Cooperation and Development), Spain is one of the OECD States with more graduates in Medicine, 13 per 100,000 inhabitants, many more than neighboring countries such as Greece, France, Italy, Germany, Belgium and the United Kingdom. The volume of annual graduates is the result of decisions made by governments (numerus clausus) when approving the number of students admitted to the Medical Universities of each country. Specifically, in Spain, in 2015, 6,053 doctors graduated. Today we are talking about 7,000 graduates who are licensed annually in any of the 42 medical Universities in Spain. Many institutions (World Health Organization, Spanish General Council of Official Associations of Physicians, National Associations of Medicine Deans) recommends that fewer graduates of these faculties come out each year, since the creation of the new faculties will increase the funnel that is produced in the MIR exam, forcing doctors to emigrate or seek another profession. Spain is always in the first places of the world ranking of medical schools by number of inhabitants ( figure 5). This fact, far from intimidating or reconstructing the political decision-makers of the concessions, has given wings to continue increasing the number of faculties. In fact, some more new schools are about to be opened soon in Navarra, Alicante and Bilbao.

To summarize, a glimpse of last year MIR examination. A total of 6,513 MIR positions for 14,466 graduates that applied to the exam. 9,923 of them had studied in Spanish Universities. The exam was taken by 13,241 (9,332 Spanish Universities) and passed it 11,289 (8,754 Spanish Universities). Of the 6,513 places awarded, 5,919 (90.8%) were awarded to MIR from Spanish Universities (67.6% of those who passed the test) and 596 (9.2%), the rest to MIR from non-Spanish Universities. Finally, a total of 2,837 who had studied in Spanish Universities, 32.4% of those who passed the test, have not obtained a place in this call. The total number of Spanish graduates without MIR position is now close to 4.000.

## Take Home Messages

• Since the demands to study Medicine is so high, most people with interest stay out of the process, since, at least in Spain, only the high school students with the best records are able to enter into a School of Medicine.
• Becoming a doctor is more than the study of a University Degree for 6 years. They need also a postgraduate specialization (MIR), in Hospitals of the National Health System, as a necessary step in order to be able to practice Medicine, either in public or private institutions.
• During the last years. there has been a decrease in the number of specialization places offered without a similar reduction in the number of undergraduate positions. Moreover, the number of medical schools has not stopped growing and places Spain as one of the countries with the highest ratio of Medical Schools and Students per inhabitant.
• As of November 2018, around 4,000 medical graduates cannot access specialized health training and may be forced into unemployment or emigration.
• While postgraduate specialization positions may have reached a maximum, the lack of a national planning and the continuing opening of new medical schools is risking the obligatory postgraduate training that all medical graduates need.

## Notes On Contributors

Joaquín García-Estañ is Professor of Physiology and currently is the Secretary of the Center for Studies on Medical Education of the University of Murcia. Orcid number: 0000-0002-7243-0240
