# Supplementary material for: Studying Medicine and being a doctor in Spain
Source: MedEdPublish (2016). 2018 Dec 7;7:276. [Version 1] doi: 10.15694/mep.2018.0000276.1 (PMC10711972; doi:10.15694/mep.2018.0000276.1)
Supplement: Peer review history

# Reviewer report: Reviewer response for version 1

**DOI:** 10.21956/mep.19728.r29549

**Published:** 2018-12-22

**Authors:** BALAJI ARUMUGAM

**Affiliations:** 1 TAGORE MEDICAL COLLEGE AND HOSPITAL

This review has been migrated. The reviewer awarded 4 stars out of 5 Thank you very much for request to review this article. The extensive literature review on the access and admission process was clearly explained in this article. The attitude of entering into medical schools among the teens and the opportunities to access to the each level was really admiring. The introduction started with process of Spanish educative system, then progressed with access and admission procedures, principles of equality, merit and ability, registration procedures for admission to medical schools, cost of medicine, and access to postgraduate training and concluded with medical demography and world ranking according to population ratio. The narrator of this article has written the article in a very nice comprehensive way that everyone can understand it clearly. Well done authors.

Reviewer Expertise:

NA

No decision status is available

# Reviewer report: Reviewer response for version 1

**DOI:** 10.21956/mep.19728.r29548

**Published:** 2018-12-15

**Authors:** sathyanarayanan varadarajan

**Affiliations:** 1 SRM Medical College, SRM University

This review has been migrated. The reviewer awarded 4 stars out of 5 This is a nice article describing “Studying Medicine and being a doctor in Spain” in which the author discusses about the process of accessing the University from Baccalaureate and Vocational Training, Procedure of admission to a Public Medical School and private universities of Spain including postgraduate training, the governing principles involved in the admission process, reservation categories in the medical school and the cost of studying Medicine in Spain.He feels that the entry of students selected solely for their excellence in high school, without taking into account of other factors such as vocation care. He has suggested including examination of clinical skills and communications, a kind of OSCE that all graduates would have to pass for qualifying entry into postgraduate training.He has also highlighted the various medical specialties positions offered there for postgraduate specialization in 2018, Number of specialization positions awarded to medical graduates from the total number of graduates that took the MIR exam and World ranking of countries ordered by Medicine Schools to population ratio with statistical data.The author concludes by saying that the lack of a national planning and the continuing opening of new medical schools is risking the obligatory postgraduate training that all medical graduates need.This article is a Bird’s eye view of studying medicine in Spain and will be useful for policy makers and administrative personnel involved in medical education.

Reviewer Expertise:

NA

No decision status is available
